# A hybrid optimization approach for intelligent manufacturing in plastic injection molding by using artificial neural network and genetic algorithm

**DOI:** 10.1038/s41598-023-48679-0

**Published:** 2023-12-09

**Authors:** Mohamed EL Ghadoui, Ahmed Mouchtachi, Redouane Majdoul

**Affiliations:** 1 Laboratory of Structural Engineering, Processes, Intelligent Systems and Informatique in ENSAM, Hassan 2 University, Casablanca, Morocco; 2 Laboratory of Complex Cyber Physical Systems in ENSAM, Hassan 2 University, Casablanca, Morocco

**Keywords:** Chemical engineering, Mechanical engineering

## Abstract

This study presents a novel hybrid optimization approach for intelligent manufacturing in plastic injection molding (PIM). It focuses on globally optimizing process parameters to ensure high-quality products while reducing cycle time, material waste, and energy consumption. The method combines a backpropagation neural network (BPNN) with a genetic algorithm (GA) and employs a multi-objective optimization model based on design of experiments (DoE). A BP artificial neural network captures the relationship between optimization goals and process parameters. Leveraging the genetic algorithm, it effectively optimizes process parameters for achieving global optimization goals. The case study involves a polypropylene product, considering dimensional deviation, weight, cycle time, and energy consumption during the PIM cycle. Design variables include melt temperature, injection velocity, injection pressure, commutation position, holding pressure, holding time, and cooling time. The results demonstrate that this approach efficiently adjusts process parameters to meet quality standards, significantly reducing raw material consumption (2%), cycle time (12%), and energy consumption (16%). This offers substantial benefits for companies in highly competitive markets demanding swift adoption of smart production methods.

## Introduction

### Process injection molding PIM

Plastic injection molding stands as a ubiquitous and indispensable technique in the production of plastic parts, wielding its influence across an array of industries such as automotive, electronics, medical, sports and recreation, construction, and consumer goods. The Handbook of Plastic Materials by Charles A. Harper^1^ meticulously delineates this versatile manufacturing process, whereby both thermoplastic and thermoset materials are transformed into an array of products, each tailored to distinct end applications. As this age-old method perseveres in the modern industrial landscape, the quest for superior quality, efficiency, and sustainability has become paramount.

### Challenges in plastic injection molding

**Figure 1:**
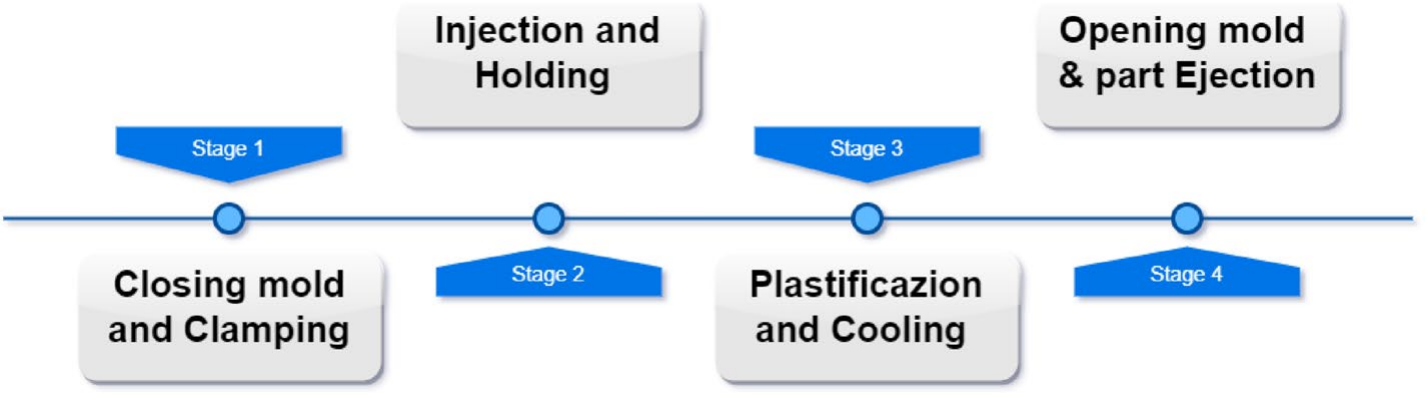
Stages description in injection cycle.

The main principle of injection molding (Fig. 1) revolves around the utilization of high pressure to shape molten plastic material. This molten material is derived from subjecting plastic to heat through the orchestration of rotating screws and heater bands, following which it is precisely injected into temperature-controlled molds, filling the cavities within. The process does not end here; it extends to the imposition of holding pressure to solidify the plastic, sealing injection gates, and concludes with the ejection of the finished part, setting the stage for the next cycle of production. In the realm of industries, it serves, quality in the injection molding process is predicated upon three vital facets:

1. Product weight consistency: it necessitates adherence to stringent tolerance limits for maintaining product integrity and performance. Deviations beyond acceptable boundaries can potentially result in performance issues or assembly complications.
2. Product size and dimension precision: accuracy, stability, deviation, and tolerance collectively contribute to achieving the desired dimensions for molded parts. Strict conformity to specifications ensures seamless integration within the intended application
3. Defect minimization: an array of defects, from warping and flash to sink marks and air traps, poses a threat to both aesthetics and functionality. Addressing these issues is crucial for upholding customer expectations and product performance.

Customer satisfaction hinges on the fulfillment of these quality requirements. However, in the face of intensified competition, escalating raw material costs, and rising energy expenditures, manufacturers are under increasing pressure to optimize their production processes. This optimization must navigate a delicate balance between sustaining quality while concurrently reducing production time, conserving raw materials, and optimizing energy consumption. Striking this equilibrium holds the key to bolstering competitiveness, enhancing profitability, and delivering cost-effective products to the market.

### Energy consumption and process complexity

It is important to acknowledge that plastic injection molding is energy-intensive, primarily due to the high-pressure prerequisites during the injection and holding phases. These phases demand substantial mold clamping force, and the plasticization phase necessitates significant heating power to effectively melt the plastic material, culminating in substantial energy consumption. Moreover, the complexity of the injection molding process is further compounded by the multitude of machine setting parameters that must be configured precisely. Achieving optimal adjustments is an intricate endeavor, especially when dependent on traditional trial-and-error methods. This approach often results in significant time, raw material, and energy wastage during the process setup and implementation.

### The imperative for new techniques

To address these challenges, it is imperative to explore novel techniques that promise holistic optimization for both customer satisfaction and producer profitability. The goal is to infuse intelligence, sustainability, and profitability into the manufacturing process. By incorporating advanced optimization algorithms and smart process control systems, the injection molding process can be streamlined, eliminating inefficiencies, reducing energy consumption, and optimizing raw material usage. This approach ensures the fulfillment of customer quality requirements while concurrently augmenting the overall sustainability and profitability of the production process to ensure companies perinity in a competitive and unstable market.

### Literature review

Research work in the field of plastic injection molding has continued to appear in order to solve the problems linked to this process, which is considered to be very important and complicated due to its variety in terms of the product shapes and sizes obtained, the materials used, the requirements requested, the industrial sector of applications served. The effective optimization of the injection molding process has been and still remains the most sought after objective and the major concern of researchers and industrialists in new context of industry 4.0^2^. To achieve this goal, different approaches and methods have been used by researchers through traditional tools^3^ and advanced methods using artificial intelligence.

However, A large number of research studies have sought to optimize the injection molding process by employing hybrid solutions, which combine Artificial Neural Networks (ANN) with various optimization algorithms such as Genetic Algorithms (GA)^4–10^, Non-dominated Sorting Genetic Algorithm II (NSGA-II)^11^, Particle Swarm Optimization (PSO)^12^, Simulated Annealing Algorithm (SAA)^13^, and Self-Organizing Maps (SOM)^14^.

Moayyedian et al.^15^ conducted a study to identify the most influential parameters among filling time, cooling time, pressure-holding time, and melt temperature in addressing three common defects in injection molding: short shot, shrinkage, and warpage. They employed the Taguchi method and combined it with an ANN and optimization algorithms. Results emphasized that filling time and pressure-holding time were the most crucial parameters affecting end-product quality. Guo et al.^16^ harnessed training data from finite element (FE) simulations to construct prediction models for warpage of microcellular foaming material. Their study compared three methods: BP neural network, GABP neural network, and PSOBP neural network. PSOBP was found to be the most precise prediction model for warpage. Optimization through genetic algorithms led to a significant reduction in warpage. Bensignth et al.^12^ investigated the injection molding of a bi-aspheric lens using PMMA material, aiming to minimize volumetric shrinkage for optical quality. They employed a hybrid ANN-PSO technique to predict optimal process parameters and achieved an optical power of 27.73 Diopter, significantly reducing spherical aberrations. Tsai and Luo^5^ integrated ANN and GA to create a reverse injection molding model for optical lens shape accuracy. Significant factors affecting the desired accuracy were determined, leading to a successful combination of ANN and GA that improved lens shape accuracy. Chen et al.^6^ introduced an optimization system combining the Taguchi method, analysis of variance, signal-to-noise ratio, BPNN, and genetic algorithms to enhance plastic injection molding (PIM). The system not only met quality specifications but also improved the stability of the PIM process.

For work devoted to the study and optimization of energy consumption in the injection molding process, Mianehrow et Abbasian^17^ followed the specific energy consumption profile of six hydraulic injection molding machines to assess the effect of different machine and process parameters and to find energy saving opportunities. Their results showed that throughput and total cycle time have the greatest impact on the specific energy consumption of the process. This effect becomes more significant as the peak power of the machine is greater.

Huszar et al.^18^ proposed a sustainable injection molding based on good choice of material and gate location to reduce warpage and injection pressure. The proposed method studied firstly the impact of four gate locations and four different materials on desired targets by using simulation software. Secondly the results obtained have been experimentally tested in part produced in the fiber-filled PP with a gate location judiciously chosen giving a successful reduction of warpage and injection pressure and then allow to reduce the production waste and energy consumption for defect-free sustainable manufacturing.

Yin et al.^7^ took into consideration in their optimization study the minimization of energy consumption by reducing the parameters that consume more energy by integrating it into the optimization constraints of the algorithm used. The main remark is that this energy optimization is not quantifiable to judge its importance.

### Importance of smart production

The literature review reveals that most optimization studies in injection molding focus on exploring the influence of process parameters on part quality. Researchers have obtained satisfactory results by employing hybrid techniques to optimize both single and multiple objectives of the desired product quality to satisfy customer requirements. However, there is a lack of attention given to the implications on production cost, including production time, energy consumption, and raw material usage. Considering the intense competition between companies and the rising costs of raw materials and energy, it becomes imperative to address these aspects.

Intelligent manufacturing emerges as the key solution to tackle this challenge by providing optimal process parameter values for global optimization and sustainable production. By integrating design of experiments and a smart hybrid technique combining artificial neural networks (ANN) and genetic algorithms (GA), this paper aims to achieve multi-objectives modeling for global optimization to satisfy both customers and producers. Although this technique has been previously explored by researchers for the optimization of the injection molding process, its use has been restricted to compliance with the quality requirements requested by customers without worrying about the impacts on production. The major contribution of this work consists of demonstrating the effectiveness of the use of this hybrid method in a context of global multi-objective optimization combining quality and production requirements that are often contradictory and then requires the search for optimal solutions. ensuring a reasonable compromise. A case study was retained as technical support to apply the method and verify its effectiveness for an overall optimization of four objectives illustrating both quality and production requirements. A design of experiments was executed based on seven most important adjustment parameters according to experts in the field and previous work. The results obtained will be tabulated to build a training dataset for the artificial neural network to develop the most representative model of the case study. This model will be combined after validation with a genetic algorithm to find an optimal solution of the adjustment parameters for global optimization of all the objectives retained.

## Optimization method and experimental setup.

### Plastic part and material

**Figure 2:**
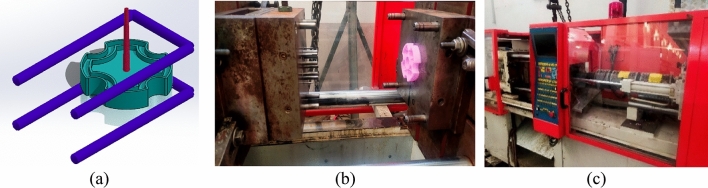
Part (**a**), mold (**b**) and machine (**c**).

The injected part used as a support for this study and represented in Fig. 2a is related to agriculture sector. The mold (Fig. 2b) is a tool with a single cavity and sprue as a feed system. The cooling system consisted of cooling channels to maintain the required mold temperature. Hydraulic injection molding machine (MIR MPO 50 T Fig. 2c) having technical characteristics presented in Table 1, was used in this study for molding of plastic part.

**Table 1:** Technical data of injection molding machine.

Properties	Unit	Value
Screw diameter	mm	34
Screw rapport L/D	mm	20
Shot size	cm^3^	136
Injection weight (PS)	g	120
Injection pressure hydraulic	Bar	140
Injection speed	cm^3^/s	70
Toggle stroke	mm	360
Space between tie bars	mm × mm	275 × 275
Min–Max. mold height	mm	160–520
Clamping force	KN	500
Energy absorption (average)	KWh	8

Polypropylene copolymer of grade 49MK45 procured from SABIC has been used for this study. The properties of the material are given in Table 2.

**Table 2:** Properties of polypropylene material.

Properties	Unit	Value (1)	ASTM method
Melt flow rate @ 230°C & 2.16 kg load	g/10 min	21	D 1238
Density @ 23°C	g/cm^3^	0.905	D 792
Vicat softening point	°C	151	D 1525B
Heat deflection temperature @ 455 kPa	°C	105	D 648
Processing conditions			
Barrel temperature range:	°C	200–280	
Mold shrinkage	%	1.2–2.5	
Mold temperature	°C	15–65	

### Experimental design

#### Inputs variables

The input variables in this study are chosen based on literature of previous studies, feedback and experimental knowledge from experts and engineers. The input process parameters of injection molding are found to be melt temperature, injection velocity, injection pressure, cooling time, holding time, position of commutation and holding pressure. The range of each process input variable in addition to the levels used in this study are given in Table 3.

**Table 3:** Injection molding input process parameters and their range and levels.

N	Variable name	Variable symbol	Range	Unit	Level 1	Level 2	Level 3
1	Melt temperature	Tmelt	220–240	°C	220	230	240
2	Injection velocity	Vinj	30–70	%	30	50	70
3	Injection pressure (hydraulic)	Pinj	40–80	Bars	40	60	80
4	Cooling time	tcool	20–40	Seconds	20	25	30
5	Holding time	th	1–10	Seconds	1	5	10
6	Commutation position	PC	10–15	mm	10	12	15
7	Holding pressure (hydraulic)	Ph	10–25	Bars	10	20	25

#### Outputs variables

The output parameters of injection molding are dimensional deviation, weight, production cycle time and energy consumed. The required output parameters of the injection molding of the plastic part used in this study are given in Table 4.

**Table 4:** Required output parameters and measuring instruments used.

	Optimizing objectives	Unit	Required values	Measuring instruments
1	Dimension and deviation	mm	32 ± 0.02	Digital caliper 0.01mm (Fig. 3a)
2	Weight	Grams	To minimize	Precise Electronic Scale 0.1mg (Fig. 3b)
3	Cycle time	Seconds	To minimize	Machine of injection timer
4	Energy consumed in cycle time (consumed per piece)	Kwh	To minimize	Power network meter Lovato electric DMG 800 (Fig. 3c)

**Figure 3:**
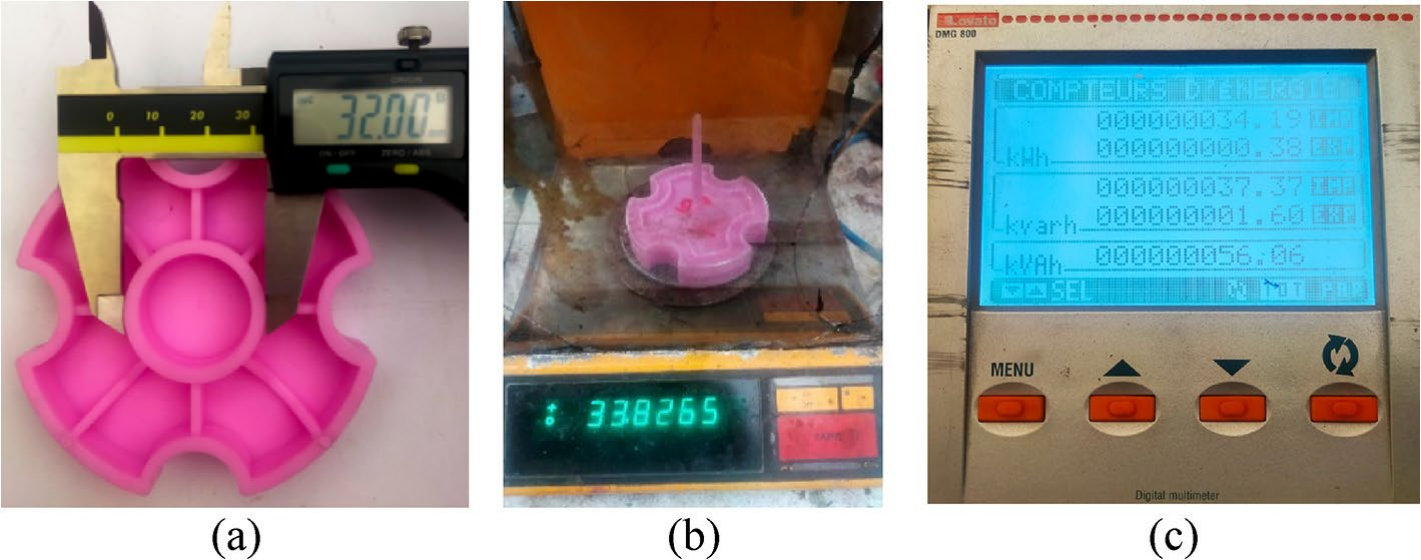
Measuring instruments: digital caliper (**a**), precise electronic scale (**b**) power network meter (**c**).

These input–output parameters are used for modeling the part injection molding process by using Artificial Neural Network. The training data of the network are obtained by running an experimental design based on an orthogonal table (L27)^3^. The input variables can be introduced and adjusted within the range as given in Table 3. Output parameters were measured and replicated five times by using instruments mentioned above in Table 4. The molded samples of part were tested for their dimensional quality after 24 h.

#### Design of experiment results

The results of input–output of the part molded in the injection molding experiment are given in Table 5. They are employed in the next paragraphs for the analyzing, modeling, and optimization.

**Table 5:** Experimental data Input–output parameters for ANN modeling.

Run	Input parameters							Output parameters			
	Tmelt	Vinj	Pinj	tcool	Th	Pc	Ph	Weight	Cycle time	Dimensional deviation	Energy consumed
1	220	30	40	20	1	10	10	34.26	31.58	0.134	0.064
2	220	30	40	25	5	12	20	35.31	40.58	0.042	0.084
3	220	30	40	30	10	15	25	36.41	50.58	0.074	0.105
4	220	50	50	20	1	10	20	34.08	30.58	0.138	0.060
5	220	50	50	25	5	12	25	35.26	39.58	0.016	0.079
6	220	50	50	30	10	15	10	36.02	49.58	0.066	0.102
7	220	70	80	20	1	10	25	33.98	30.11	0.148	0.062
8	220	70	80	25	5	12	10	34.90	39.11	0.030	0.081
9	220	70	80	30	10	15	20	35.88	49.11	0.058	0.101
10	230	30	50	20	5	15	10	35.07	35.53	0.076	0.076
11	230	30	50	25	10	10	20	35.85	45.53	0.044	0.096
12	230	30	50	30	1	12	25	34.06	41.53	0.048	0.087
13	230	50	80	20	5	15	20	34.89	34.51	0.090	0.074
14	230	50	80	25	10	10	25	35.90	44.51	0.030	0.093
15	230	50	80	30	1	12	10	33.86	40.51	0.050	0.081
16	230	70	40	20	5	15	25	35.01	34.18	0.046	0.071
17	230	70	40	25	10	10	10	35.71	44.18	0.026	0.091
18	230	70	40	30	1	12	20	33.79	40.18	0.038	0.083
19	240	30	80	20	10	12	10	35.67	40.40	0.024	0.083
20	240	30	80	25	1	15	20	33.89	36.54	0.110	0.074
21	240	30	80	30	5	10	25	34.86	45.57	0.008	0.094
22	240	50	40	20	10	12	20	35.53	39.48	0.066	0.086
23	240	50	40	25	1	15	25	33.87	35.48	0.130	0.073
24	240	50	40	30	5	10	10	34.60	44.48	0.032	0.092
25	240	70	50	20	10	12	25	35.63	39.09	0.102	0.079
26	240	70	50	25	1	15	10	33.56	35.09	0.204	0.073
27	240	70	50	30	5	10	20	34.54	44.09	0.062	0.090

### Taguchi design analysis

#### Impact factors

Response table for S/N ratio is used in Minitab software to give the rank for each factor based on the Delta value which is the difference between the highest and lowest average response values. The results in Table 6 presented above give the Rank that indicate the relative effect of each factor on the response. For product weight, the main impact factor is the holding time which the effect is considerably larger than all other factors. For the cycle time, it is logically impacted by temporal factors which are the holding and the cooling times. Regarding the dimensional deviation used as a product quality index, all factors retained in this study have a considerable influence on this objective with a greater effect for holding time, position of commutation and cooling time, compared to the other remaining factors. The energy consumption is affected by the temporal factors and then presents a correlation with cycle time.

**Table 6:**
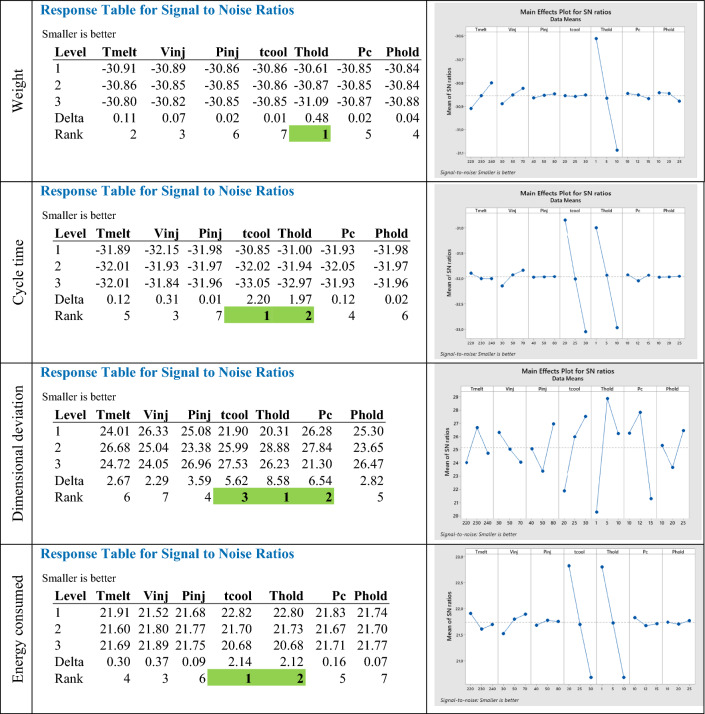
Responses table and main effects plots for S/N Ratio.

**Figure 4:**
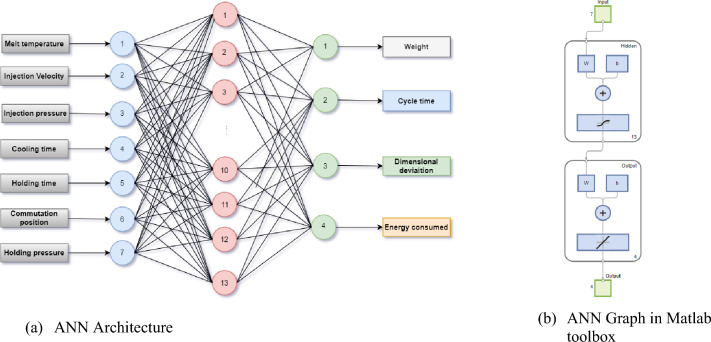
Multilayer ANN for process mapping of part injection molding.

The main effects plots (S/N ratio) are used to show how each factor affects the responses characteristics as we ca see in Fig. 4. In addition, the analysis of thus graphs shows the direction of variation of the objectives depending on the factors studied. In this context, the factors allowing the improvement of the quality objective, by a reduction in the dimensional gap mentioned above, must be increased. consequently, the cycle time being lengthened, the energy consumption increases as well as the weight of the part. The search for effective methods and techniques, allowing the obtaining of an optimal solution ensuring the requested quality by reducing cycle time and saving energy and material consumption, is a primary requirement for producers.

#### Optimal configuration for dimensional deviation using S/N ratio

In this section the signal-to-noise ratio (S/N ratio) is used to identify the levels of control factor settings that minimize the main quality index required by costumer which is the dimensional deviation. The optimal parameters configuration giving in Table 7 is then choosing in level for each factor when S/N ratio is maximal as shown in mains effect plot of S/N ratio above. This optimal configuration does not appear in the experimental design carried out but it can be replaced by test 21 which is almost identical with a difference in melt temperature value which is a factor with limited effect (Rank 6) on the response as shown response table above.

**Table 7:** Optimal configuration for dimensional deviation using S/N ratio.

	Tmelt	Vinj	Pinj	tcool	th	Pc	Ph	Weight	Cycle time	Dim dev	Energy consumed
Optimal configuration S/N Ratio (run N 21)	240	30	80	30	5	12	25	34.86	45.57	0.008	0.094

### ANN model

#### Architecture neural network

**Figure 5:**
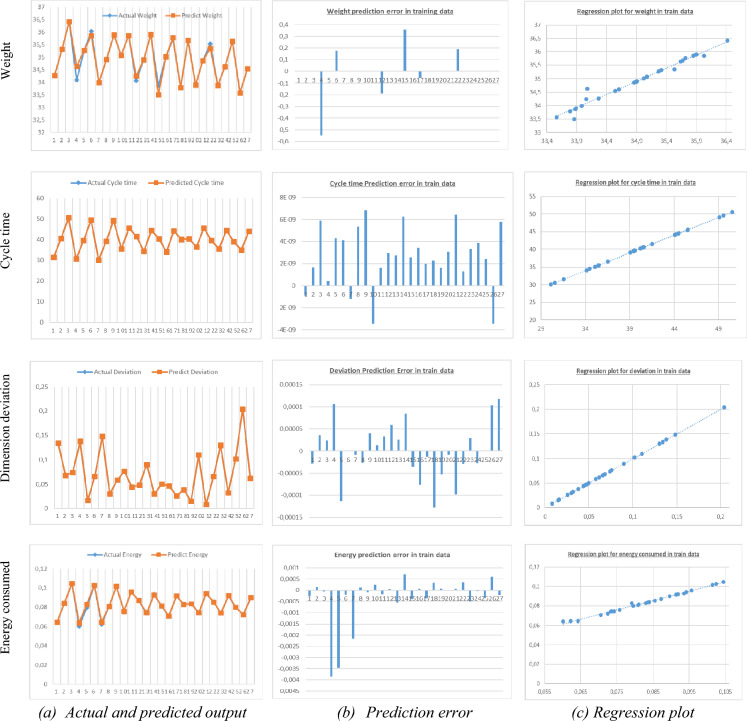
Results plots of ANN model developed for process mapping of part injection molding in training data.

Matlab 2022a software and Neural Network toolbox were used to develop the desired model of the part injection molding process. The number of input layer neurons is seven, the number of neurons in the output layer is four corresponding to targets followed. To avoid overfitting issue^3,19^, the architecture of ANN to use has been simplified to one hidden layer in the goal to increase its ability for generalization^20^ mainly because of the use of limited amount of data. The choice of the number of neurons in this layer is based on a practice rule that consists to retain a value less than twice the number of input variables^12^. The optimal value is obtained after tuning hyper-parameters step and it is finally set to 13. The activation function of hidden layer neurons was tan-sigmoidal and for output layer is a pure line functions. Three learning algorithms have been used which are Levenberg–Marquardt (LM), Bayesian regularization (BR) and scaled conjugate gradient (SCG). LM was especially developed for faster convergence in backpropagation algorithms. Essentially, BR, that naturally incorporates regularization, has an objective function that includes a residual sum of squares and the sum of squared weights to minimize estimation errors and to achieve a good generalized model^21^. SCG is an efficient optimization algorithm primarily applied in training neural networks. Therefore, the developed multilayer Neural Network is (7-13-4) as shown in Fig. 5 on representation graph (Fig. 5a) and in Matlab neural network tool (Fig. 5b).

#### ANN performances evaluation.

The developed ANN was trained with all experimental data consisting 27 sets, (100% of total dataset) to keep the orthogonality characteristic of table used to ensure the good accuracy of the model. Additional experiments data consisting of 5 sets were used for model testing. The performance of the developed ANN was evaluated through coefficient of determination (R^2^), mean squared error (MSE), root mean squared error (RMSE) and relative percent deviation (RPD) as given below.

R^2^: Regression R values measure the correlation between outputs and targets. An R^2^ value of 1 means a close relationship, 0 a random relationship.

1 $$R^{2}=1-((\sum {\left(yi-\widehat{y}i \right)}^{2}/\sum {\left(yi-\overline{y } \right)}^{2})$$

MSE: mean squared error is the average squared difference between outputs and targets. Lower values are better. Zero means no error.

2 $$Mse=(1/n)*\sum (yi-\widehat{y}i )^{2}$$

RMSE: root mean squared error is the racine of the average squared difference between outputs and targets. Lower values are better.

3 $$RMSE=\sqrt{((1/n)*\sum (yi-\widehat{y}i )^{2}}$$

4 $$\mathrm{RPD\%}:\mathrm{ relative \, percent \, deviation \;RPD\%}=(100/n)((\sum |(yi-\widehat{y}i) |/|yi|)$$

where n is the total number of data sets, yi is the ith actual data point value, $\widehat{y}$ i is the ith predicted data point value and $\overline{y }$ is the mean of actual output values.

#### Training ANN multi-responses model and comparison

The data and results of training the neural network to develop a multi-objectives model for process parameters optimization are given in Table 8 below. The comparison of the overall result between the three algorithms used is based on the MSE and the R2 cited above. As we can see on this table, the best model is model 2 which uses Bayesian regularization as a training algorithm.

**Table 8:** Results comparison after training and testing of the three models.

	Algorithm used	Hidden nodes	Data split	Training data		Testing data	
				MSE	R^2^	MSE	R^2^
Model 1	Levenberg–Marquardt	13	100-0-0	2.626 e−8	1	0.374	0.999
Model 2	Bayesian regularization	13	100-0-0	8.641 e−8	1	0.033	1
Model 3	Scaled conjugate gradient	13	100-0-0	1.011 e−4	1	0.872	0.999

### Model responses evaluation in training data

**Figure 6:**
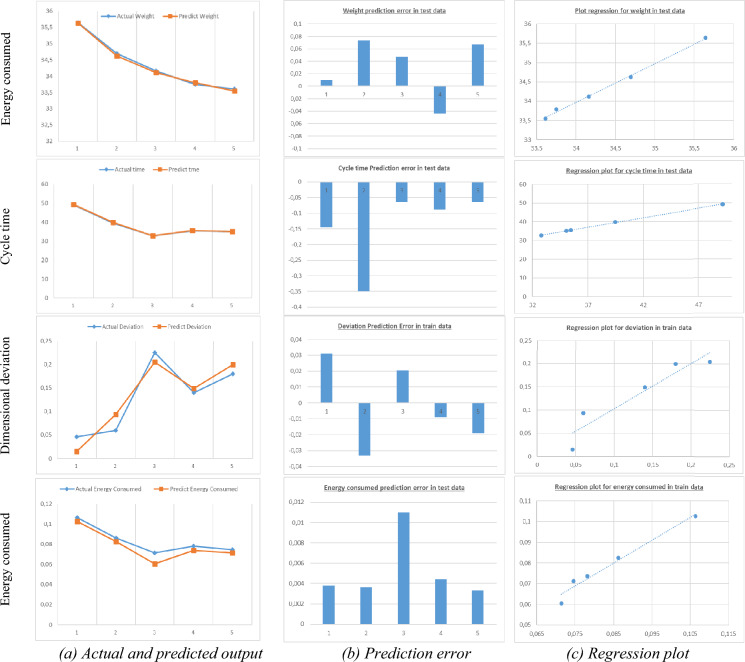
Results plots of ANN model developed for process mapping of part injection molding in testing data.

In this paragraph the results of the performance of model 2 retained are given in detail for the four responses adopted in this study. They are plotted in Fig. 6 and tabulated in Table 9 according to the criteria of the MSE, MAE, R2 and RDP%. It is observed that the training results are very satisfactory to the point of fearing overfitting which requires to check the model on additional test data.

**Table 9:** Prediction performance parameters of developed ANN for training data set.

Part output parameters	R^2^	MSE	RMSE	RPD, %
Weight	0.985	0.019	0.140	0.16
Responses				
Cycle time	1	1.14 e−17	3.75 e−9	8.03 e−9
Dimensional deviation	0.999	3.89 e−9	6.24 e−5	0.15
Energy consumed per cycle	0.996	1.25 e−6	0.0011	0.79

#### Model responses evaluation in testing data

Five additional experiments, giving in Table 10, have been conducted for the use in the testing accuracy of the model obtained after training step. Based on the same evaluation criteria, the results of this testing step for all objectives followed are illustrated on Table 11 and represented graphically in Fig. 6. As we can see, the model is accurate enough to be used for optimization by the genetic algorithm in the next paragraph in search of optimal process parameter values.

**Table 10:** Additional testing data.

Run	Input parameters							Output parameters			
	Tmelt	Vinj	Pinj	tcool	Th	Pc	Ph	Weight	Cycle time	Dimensional deviation	Energy consumed
1	240	70	80	30	10	15	20	35.64	49.15	0.046	0.106
2	240	50	50	25	5	12	15	34.69	39.47	0.060	0.086
3	240	25	40	20	2	10	10	34.16	32.77	0.224	0.071
4	240	50	40	25	1	15	20	33.74	35.48	0.1401	0.078
5	240	70	60	25	1	15	10	33.610	35.06	0.180	0.074

**Table 11:** Prediction performance parameters of the developed ANN for testing data set.

Part output parameters	R^2^	MSE	RMSE	RPD%
Weight	0.998	0.0028	0.053	0.14
Cycle time	0999	0.0318	0.178	0.36
Dimensional deviation	0.940	0.0005	0.024	29.7
Energy consumed per cycle	0.983	3.58e−5	0.006	6.65

#### Results of output parameters analysis

**Figure 7:**
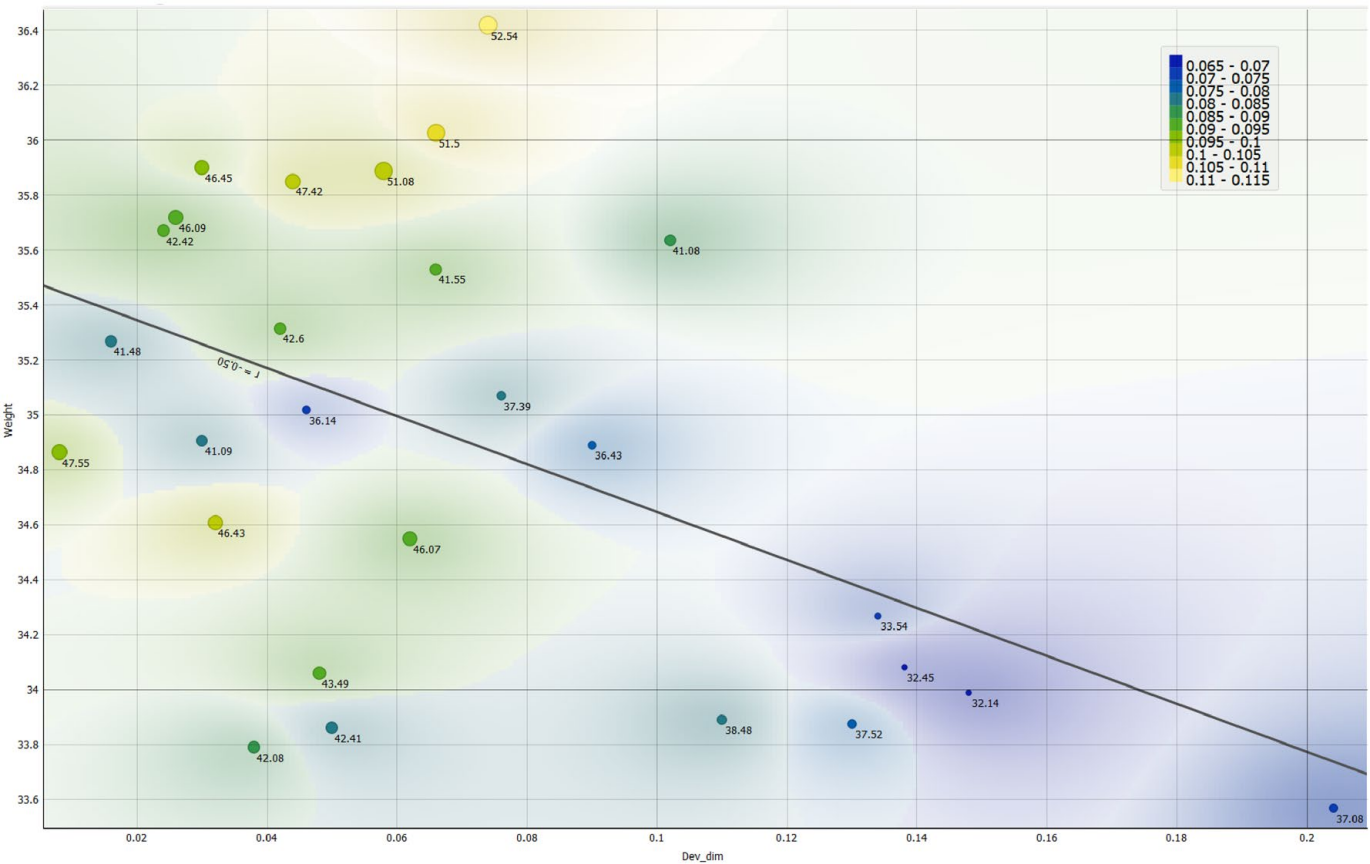
Analysis plot of the relationships between the four objectives monitored.

In Fig. 7, the dimensional deviation is plotted against the mass of the part, while the energy consumption is represented by colored areas ranging from yellow to blue, indicating different levels of consumption. The cycle time is also indicated and labeled for each data point on the plot. From the graph, it is evident that improving the quality of the product by reducing dimensional deviation comes at a cost. This cost is reflected in increased mass, longer cycle times, and higher energy consumption. However, there are certain areas on the graph that indicate potential actions for achieving a global optimization of the objectives.

This analysis highlights the complex and often contradictory relationship between customer product quality and the parameters that need to be optimized from a producer's perspective. It is not feasible to achieve this global optimization manually through setters alone. Therefore, the developed ANN model, combined with the use of GA as a hybrid technique, becomes crucial. This combination allows for a compromise to be made to globally optimize the injection molding process parameters for the product. By integrating the ANN-GA hybrid technique, the injection molding process can strike a balance between meeting customer quality requirements and optimizing the parameters that affect production costs such as cycle time and energy consumption. This approach enables a more comprehensive and effective optimization process, leading to enhanced efficiency, profitability, and sustainability in the production of injection-molded products.

### Process parameters optimization using genetic algorithm

#### Mathematical formulation of optimization problem

The developed ANN can be coupled to GA for optimizing the injection molding process parameters. The multi-objective optimization model can be stated as follows:

Find: Tmelt, Vinj, Pinj, tcool, th, PC, and Ph

5 $$\mathrm{Min f}(\mathrm{x}) = \{\mathrm{Dimensional \;deviation},\mathrm{ Weight},\mathrm{ cycle \;time \;and \;Energy \;consumed}\}$$

Subjected to constraints:

6 $${22}0 \le {\text{Tmelt}} \le {24}0$$

7 $${3}0 \le {\text{Sinj}} \le {7}0$$

8 $${4}0 \le {\text{Pinj}} \le {8}0$$

9 $${2}0 \le {\text{tcool}} \le {3}0$$

10 $${1} \le {\text{th}} \le {1}0$$

11 $${1}0 \le {\text{Cp}} \le {15}$$

12 $${1}0 \le {\text{Ph}} \le {2}0$$

**Figure 8:**
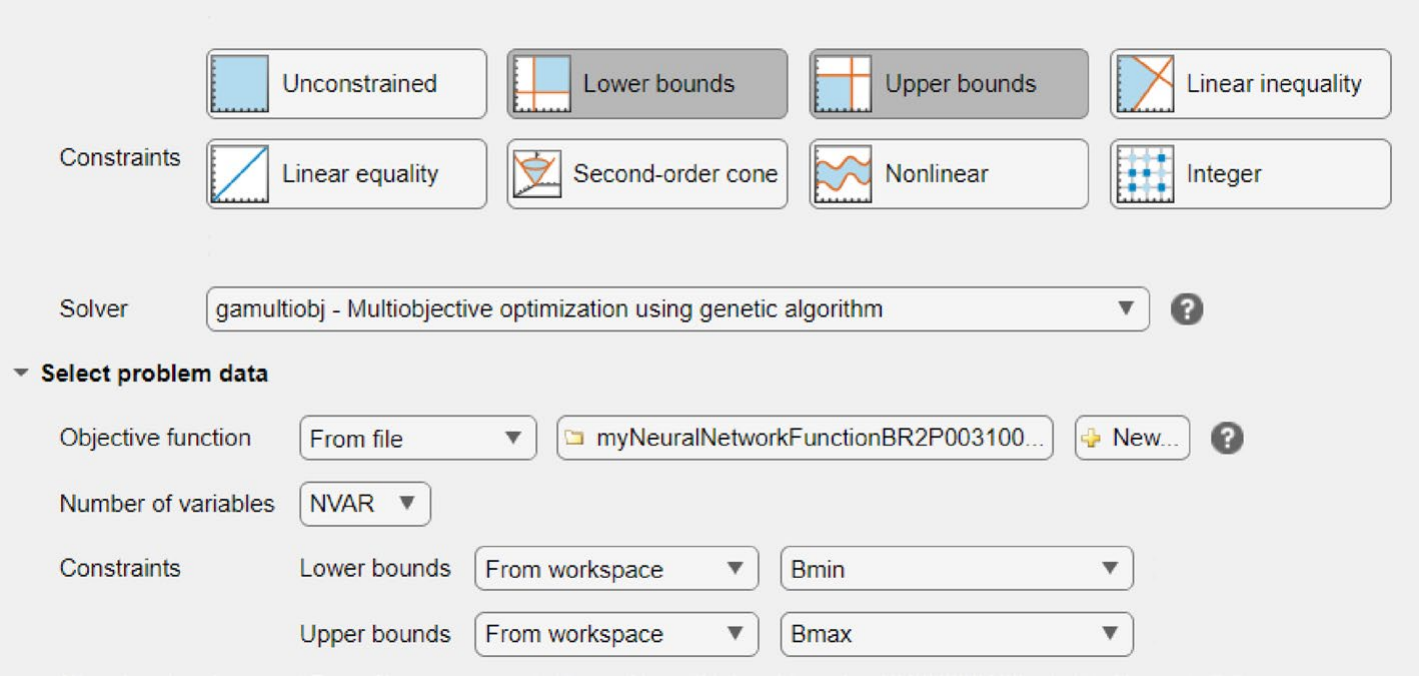
Matlab tool interface of GA for multiobjective optimization.

The implementation of problem optimization is carried out in toolbox optimization in Matlab 2022a environment. The goal is to find optimal process parameters of part injection molding process. In this stage, the developed ANN model retained (7-13-4) is transformed to Matlab function and introduced as a fitness function with number of variables and bounds as shown in Fig. 8.

#### Parameters of genetic algorithm

The main set parameters of multi-objective genetic algorithm used are:

- Population size of 200,
- Mutation probability is 0.35,
- Crossover probability is 0.8
- Maximum generation number of 100.

#### Optimization results

The optimal configuration of process parameters and their corresponding predicted outputs parameters given by genetic algorithm are tabulated in Table 12. It should be noted that the execution of the optimization is repeated several times, and the mean of the values has been retained for each parameter.

**Table 12:** GA predicted optimum process and output parameters for injection molding process.

Run number	GA optimization process input parameters										
	Tmelt	Vinj	Pinj	tcool	th	Pc	Ph	Weight	Cycle time	Deviation	Energy per cycle
Mean	226	61	76	25	5	10	17	34.81	39.58	0.018	0.082

#### Experimental results evaluation and discussion

To evaluate the precision of the developed model and verify the effectiveness of the global optimization approach, the optimal configuration obtained from the genetic algorithm was implemented in an experimental evaluation using an injection machine. The first objective, assessing the accuracy of the model, involved comparing the output parameter values proposed by the genetic algorithm with the results obtained through experimentation. Table 13 illustrates the comparison, revealing a high level of accuracy in the model with a margin of error less than 2.5%. The second objective, evaluating the effectiveness of the method, involved comparing the optimal configuration results obtained from the genetic algorithm with the results obtained from the analysis of signal-to-noise ratio. In this comparison, the results of the configuration from the plan of experiments (Run N 21) were utilized. The effectiveness of the hybrid ANN-GA method in the global optimization approach was demonstrated through the results presented in Table 14. The results clearly indicate that the hybrid method effectively optimized the injection molding process. The dimensional quality required by the customer (± 0.02) was achieved, while simultaneously achieving a 16% reduction in energy consumption, a 2% reduction in raw material usage, and a 12% reduction in production cycle time. These findings highlight the significant improvements in sustainability and cost-effectiveness achieved through the application of the ANN-GA hybrid approach. Overall, the experimental evaluation confirmed the precision of the developed model and demonstrated the effectiveness of the proposed global optimization method. The combination of ANN and GA proved to be a valuable approach in optimizing the injection molding process parameters to meet customer quality requirements while simultaneously improving energy efficiency, raw material utilization, and production cycle time.

**Table 13:** Comparison Predicted and Experimental GA optimum process and output parameters.

Configuration	Tmelt	Vinj	Pinj	tcool	th	Pc	Ph	Weight	Cycle time	Dim dev	Energy consumed
DoE (Set n°21)	240	30	80	30	5	10	25	34.86	45.57	0.008	0.094
Proposed by GA (Exp)	226	61	76	25	5	10	17	34.19	40.54	0.018	0.081
Gain %								− 2%	− 12.4%		− 16%

**Table 14:** Comparison DoE (Set N° 21) and experimental GA optimum process parameters.

N	Tmelt	Vinj	Pinj	tcool	th	Pc	Ph	Weight	Cycle time	Dim dev	Energy consumed
GA proposed	226	61	76	25	5	10	17	34.81	39.58	0.0180	0.082
GA tested in exp	226	61	76	25	5	10	17	34.79	39.34	0.0185	0.081
Margin of error								0.1%	2.3%	2.5%	1.5%

**Figure 9:**
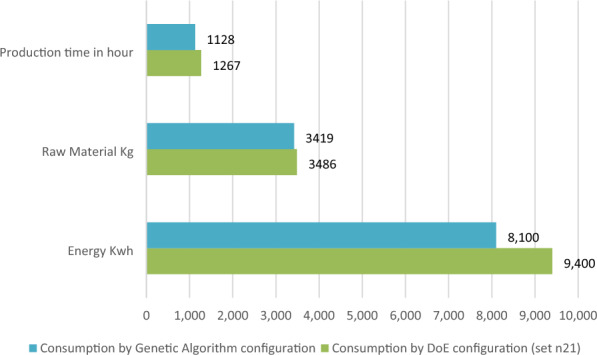
Consumption comparison graph for an order of 100,000 products.

To demonstrate the economic efficiency of the proposed technique combining ANN-GA, the production time, consumption of raw material, and energy for an order of 100,000 products (approximately equivalent to a monthly production) are presented in Table 15 and Fig. 9. The results serve as evidence of the economic benefits achieved through the implementation of the proposed technique. These results highlight the potential for cost optimization, improved production efficiency, and enhanced sustainability, all of which are crucial factors for manufacturers striving for profitability and competitiveness in the market. These results are particularly relevant for producers dealing with low-mass products that are injected using relatively small machines with a capacity of 50 tons. The table and figure provide a clear overview of the economic implications of implementing the optimized process parameters. By utilizing the ANN-GA approach, significant improvements in economic efficiency can be observed. The production time is reduced, leading to faster order fulfillment and increased productivity. Additionally, there is a notable reduction in the consumption of raw materials, resulting in cost savings and improved resource utilization. The energy consumption is also decreased, contributing to both cost savings and environmental sustainability. Overall, the combination of ANN-GA proves to be a valuable tool for achieving economic efficiency in the injection molding process. The results demonstrate the potential for cost reduction and resource optimization, making it an attractive approach for producers in various industries, especially those dealing with small-scale machines and low-mass products.

**Table 15:** Comparison experimental GA optimum output parameters according to objective required.

		Energy Kwh	Raw material kg	Production time in hour
1	Consumption by using DoE optimal configuration (set n21)	9400	3486	1267
2	Consumption by using Genetic Algorithm optimal configuration	8100	3419	1128
Gain		1300	67	139

## Conclusion

This study proposes an efficient global optimization approach for process parameters in injection molding using design of experiments (DoE), artificial neural networks (ANN), and genetic algorithms (GA). The aim is to produce a polypropylene plastic part in the industrial sector within the framework of intelligent manufacturing required by Industry 4.0. The design parameters selected are melt temperature, injection velocity, injection pressure, holding time, holding pressure, commutation position, and cooling time. The desired objectives include product weight, production cycle time, dimensional deviation, and energy consumption. The research begins by designing and conducting experiments to gather data for training and testing an artificial neural network model. This model serves as a multi-objective predictive tool for product quality and production parameters. Subsequently, a genetic algorithm (GA) is employed to determine the optimal configuration of the output parameters that satisfy the desired responses. The obtained results are then experimentally validated to assess the accuracy of the developed model, with a margin of error less than 2.5%. Furthermore, a comparison is made between the results of the experimental plan and those achieved through the proposed method. This comparison demonstrates the effectiveness of the approach, with notable gains observed in production time (12% reduction), raw material usage (2% reduction), and energy consumption (16% reduction). Additionally, the study suggests that the optimization method utilized can have a positive impact on other quality aspects of the injection molding process. Furthermore, it highlights the potential applicability of this approach in other industrial processes to address multi-objective problems and achieve smart, sustainable, and profitable production. In conclusion, this research contributes to the field of intelligent manufacturing by offering an efficient optimization method for injection molding processes. By combining DoE, ANN, and GA, it enables the attainment of optimal process parameters while considering multiple objectives and ensuring sustainable and profitable production. Finally, in future research studies, several improvements can be made to the proposed context of global optimization and notably its use on other parts and other plastic materials with more objectives and considering other parameters of the process. This will make it possible to detect the limits of the proposed method and thus to find solutions to overcome them or even to adopt other optimization techniques which will allow better generalization and thus effective control of the process and its use for parts intended for various industrial sectors.

## Data Availability

All data used in this study are included in this published article.
